# Point-of-care ultrasound used to exclude penile fracture

**DOI:** 10.1186/2036-7902-4-17

**Published:** 2012-07-13

**Authors:** Adam Ash, Joel Miller, David Preston

**Affiliations:** 1 Department of Emergency Medicine, William Beaumont Army Medical Center, 5005 North Piedras Street, El Paso, TX, 79920, USA; 2 Department of Emergency Medicine, Darnall Army Medical Center, 36000 Darnall Loop, Fort Hood, TX, 76544, USA; 3 Division of Urology, University of Kentucky, 800 Rose Street, Kentucky, KY, 40536, USA

**Keywords:** Penile hematoma, Cavernosonography, Magnetic resonance imaging, Ultrasound

## Abstract

This is a case report of a superficial penile hematoma that was difficult to distinguish clinically from a penile fracture. Such cases occur with relative frequency, and because definitive treatment is an urgent surgery, timely diagnosis is essential to avoid complications. Typical imaging modalities such as cavernosonography and magnetic resonance imaging can be invasive (cavernosonography) or time consuming (magnetic resonance imaging) and may not be readily available. Ultrasound has been used successfully in such cases, and, in this case, we used point-of-care ultrasound combined with a brief period of observation to exclude penile fracture.

## Background

Penile fracture is a well recognized yet relatively infrequent occurrence, accounting for approximately 1 in 175,000 hospital admissions [1]. The classic presentation includes post-traumatic penile pain associated with shaft deviation, eccyhmosis, and a palpable defect in the tunica albuginea usually due to trauma during sexual intercourse or masturbation [2]. Occasionally, the presentation is more subtle, and diagnosis based on history and physical examination alone is not possible. We present a case in which point-of-care ultrasound was used to exclude a penile fracture in a patient with some, but not all, of the characteristic clinical findings.

## Case presentation

### Case

**Figure 1:**
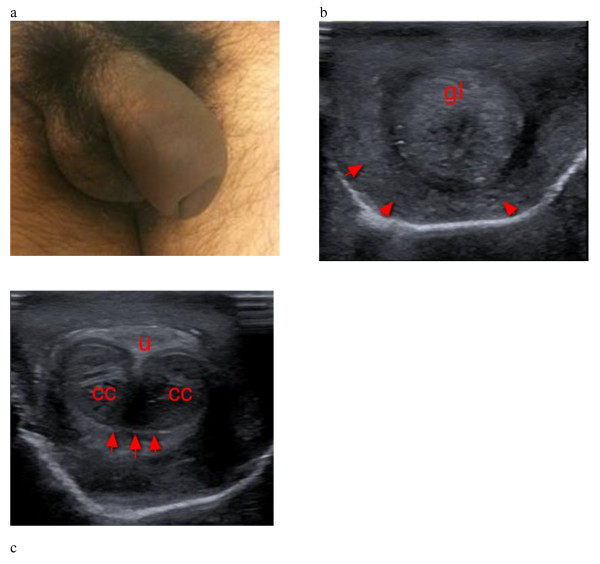
**Penile deformity, foreskin hematoma, and intact tunica albuginea.** (**a**) Penile deformity suspicious for penile fracture. (**b**) Foreskin hematoma (arrows) surrounding the glans (**gl**). (**c**) Intact tunica albuginea (arrows) surrounding the corpus cavernosum (**cc**). The urethra (**u**) appears intact.

A 29-year-old uncircumsized male presented to our emergency department approximately 30 min after being struck in the groin with the butt of a rifle. He stated that his penis was flaccid at the time of the injury and complained of pain and swelling to his penile shaft. Physical exam revealed significant edema of the penis and foreskin as well as the so-called ‘eggplant deformity’ commonly associated with penile fractures (Figure 1a). There was no palpable defect or pain to palpation over the corpus cavernosa and no testicular swelling or tenderness to palpation. Point-of-care ultrasound was performed and showed a distal foreskin hematoma surrounding the glans (Figure 1b). The cavernosa and the tunica albuginea appeared to be intact (Figure 1c). The patient was admitted to the urology service for observation overnight and discharged the following day. In follow-up, the patient’s edema resolved, and he had been able to sustain nonpainful erections without difficulty.

## Discussion

The penile body is composed of three erectile structures, two corpus cavernosa (right and left) and one corpus spongiosa (central), which contains the penile urethra. The tunica albuginea is a fibrous sheath that encapsulates all three structures. A penile fracture occurs when the tunica albuginea and corpus carvernosa are ruptured, almost always secondary to trauma [2].

Penile fracture is one of the less frequent urological traumas [3]. Patients generally report a popping sound, followed by pain and penile detumescence with the eventual development of swelling, hematoma, and penile deformity. The differential diagnosis includes injury to the dorsal penile vessels and extraalbugineal hematoma [4]. Differentiating these diagnoses from penile fracture is important clinically because fractures require surgical intervention, whereas dorsal penile vessel injuries and extraalbugineal hematomas can generally be managed conservatively. Although all three entities cause penile pain and swelling, history and physical examination are often sufficient to make this distinction. Extraalbugineal hematoma, for example, is more common with trauma to a flaccid penis, whereas fractures usually occur when the penis is erect [4]. Rupture of the dorsal penile vessels tends to occur with trauma to the erect penis, but detumescense tends to be delayed, unlike with fracture. Fractures also tend to present with palpable defects of the cavernosa that are usually painful to palpation, a finding usually not present with more superficial injury.

Atypical cases, however, do occur, and in one study, history and physical examination were inaccurate in 15% of patients [5] with suspected penile fracture. In such cases, imaging studies may be helpful to clarify the diagnosis.

Multiple imaging modalities have been used to evaluate suspected penile fractures. Cavernosonography is invasive, involves injecting a dye into the cavernosa, and has been associated with side effects including allergic reactions and priapism [1]. Magnetic resonance imaging has been used recently with reasonable accuracy but is time consuming and not always available [6]. Ultrasound has been used increasingly, and there are some older case reports describing successful diagnosis in this setting [7,8]. Recent advances in ultrasound technology (higher-frequency probes capable of identifying smaller tears and defects in the tunica albuginea and corpus cavernosa) have made ultrasound a more reliable imaging modality when penile fracture is suspected [9]. The technique consists of imaging the penile shaft with a high-frequency probe in both the transverse and horizontal planes looking for defects in the tunica albuginea, which normally appears as a hyperechoic structure surrounding the corpus cavernosa. Disruption of tunica and a hematoma are required to make the diagnosis [2].

Urethral injuries are present in approximately 20% of patients with penile fractures [8]. This diagnosis should be entertained if the patient complains of difficulty voiding or if urinanalysis shows hematuria. Although the role of ultrasound is less clear in these cases, sonographic findings suggestive of urethral rupture are a discontiguous penile urethra, air in the cavernosal bodies, and edema or hematoma of the corpus spongiosum, although urethrography will likely be required to accurately confirm or exclude the diagnosis [4].

Left untreated, penile fracture can lead to complications which include chronic pain, penile curvature, arteriovenous fistulas, and erectile dysfunction in 10% to 53% of patients [10]. Although ultrasound can be a useful complementary study when the diagnosis is unclear, its accuracy has not been reported in the literature. Because of this, positive findings should be considered suggestive, but not diagnostic, of penile fracture. Similarly, negative studies, while reassuring, cannot be relied upon to fully exclude this diagnosis. All patients with suspected penile fracture should be evaluated by a urologist urgently. Those with atypical histories, but positive ultrasound studies, should be strongly considered for urgent surgical intervention. Those with negative ultrasound studies may benefit from a brief observation period.

## Conclusion

Penile fractures occur infrequently. Although diagnosis can often be made on clinical grounds alone, dorsal penile vessel injury and extraalbugineal hematoma can mimic fractures, complicating evaluation. In cases in which the distinction is uncertain, point-of-care ultrasound is a noninvasive, time-eficient modality that may be used to further clarify diagnosis.

## Consent

Written informed consent was obtained from the patient for publication of this case report and any accompanying images. A copy of the written consent is available for review by the Editor-in-Chief of this journal.

## Competing interests

The authors declare that they have no competing interests.

## Authors’ contributions

AA saw the patient, obtained ultrasound images, performed a literature search, and wrote the manuscript. JM edited the manuscript for content and grammar, reviewed ultrasound images, and assisted with the literature search. DP evaluated the patient in the emergency department as a consultant, assisted in obtaining ultrasound images, edited the manuscript for content and grammar, and assisted with the literature search. All authors read and approved the final manuscript.
